# ACL-DUNet: A tumor segmentation method based on multiple attention and densely connected breast ultrasound images

**DOI:** 10.1371/journal.pone.0307916

**Published:** 2024-11-01

**Authors:** Hao Zhang, He Liang, Guo Wenjia, Ma Jing, Sun Gang, Ma Hongbing

**Affiliations:** 1 School of Computer Science and Technology, Xinjiang University, Urumqi, Xinjiang, China; 2 Department of Electronic Engineering, and Beijing National Research Center for Information Science and Technology, Tsinghua University, Beijing, China; 3 Cancer Institute, Affiliated Cancer Hospital of Xinjiang Medical University, Urumqi, Xinjiang, China; 4 Department of Breast and Thyroid Surgery, The Affiliated Cancer Hospital of Xinjiang Medical University, Urumqi, Xinjiang, P.R. China; 5 Xinjiang Cancer Center/Key Laboratory of Oncology of Xinjiang Uyghur Autonomous Region, Urumqi, Xinjiang, P.R. China; Memorial Sloan Kettering Cancer Center, UNITED STATES OF AMERICA

## Abstract

Breast cancer is the most common cancer in women. Breast masses are one of the distinctive signs for diagnosing breast cancer, and ultrasound is widely used for screening as a non-invasive and effective method for breast examination. In this study, we used the Mendeley and BUSI datasets, comprising 250 images (100 benign, 150 malignant) and 780 images (133 normal, 487 benign, 210 malignant), respectively. The datasets were split into 80% for training and 20% for validation. The accurate measurement and characterization of different breast tumors play a crucial role in guiding clinical decision-making. The area and shape of the different breast tumors detected are critical for clinicians to make accurate diagnostic decisions. In this study, a deep learning method for mass segmentation in breast ultrasound images is proposed, which uses densely connected U-net with attention gates (AGs) as well as channel attention modules and scale attention modules for accurate breast tumor segmentation.The densely connected network is employed in the encoding stage to enhance the network’s feature extraction capabilities. Three attention modules are integrated in the decoding stage to better capture the most relevant features. After validation on the Mendeley and BUSI datasets, the experimental results demonstrate that our method achieves a Dice Similarity Coefficient (DSC) of 0.8764 and 0.8313, respectively, outperforming other deep learning approaches. The source code is located at github.com/zhanghaoCV/plos-one.

## 1 Introduction

Breast cancer is the most common cancer type in women and one of the leading causes of female mortality globally [1, 2]. According to data from the World Health Organization (WHO), breast cancer has the highest incidence and mortality rates among female cancers worldwide. Early diagnosis and accurate treatment are crucial for achieving favorable prognosis and survival rates for breast cancer patients [3]. Currently, ultrasound imaging is a well-established tool for early assessment and diagnosis of abnormal breast conditions due to its non-invasive imaging procedure, high sensitivity, and cost-effectiveness [4, 5], Therefore, it is widely used for breast cancer screening. But we also need to know that it is primarily used as an adjunct to mammography rather than a standalone screening tool. This is because mammography is the standard screening method, and breast ultrasound is typically used to verify mammographic findings or guide biopsies, especially in patients with dense breast tissue. Therefore, Breast ultrasound is particularly beneficial for patients with dense breast tissue, as it can provide clearer images than mammography. However, due to the complex structure of the breast, standard breast ultrasound imaging is associated with high inter-reader variability and false positives, radiologists sometimes struggle to accurately locate lesions, and the heavy workload can lead to missed diagnoses and misdiagnoses, leading to unnecessary biopsies. For the rapid and effective screening of breast cancer, there is a critical Need for automated, accurate breast tumor segmentation methods to assist radiologists in diagnosing and treating breast cancer. Therefore, Computer-Aided Diagnosis (CAD) technology has emerged, bringing about significant technological breakthroughs in medical diagnosis [6] and reducing the workload of physicians. Breast masses are one of the most distinctive manifestations for diagnosing breast cancer, and their edge information reflects their growth patterns and biological characteristics. Generally, benign masses exhibit regular shapes, while irregular margins are often associated with malignancy. In other words, the accuracy of mass segmentation directly impacts the benign or malignant classification of the masses. Therefore, precise segmentation of breast tumors is of paramount importance for the classification of ultrasound images into benign or malignant categories.

Methods for breast tumor segmentation can be categorized into traditional methods and deep learning methods. Many traditional methods have been proposed for accurate breast tumor segmentation, such as active contour-based methods [7, 8], threshold-based segmentation methods [9, 10], and graph-based segmentation methods [11, 12]. In previous literature [13–15], machine learning techniques like logistic regression, random forest, and feedforward neural networks have been applied for the diagnosis of gastric cancer and breast cancer-related diseases. These traditional methods are straightforward but require extensive domain knowledge and expertise to extract color, shape, and texture features. Additionally, these methods are sensitive to noise and tend to result in over-segmentation. In recent years, due to the ever-increasing computing power and the ever-increasing amount of data available, deep learning has made significant progress in the field of medical image [16–22]. Especially, Convolutional Neural Network (CNN) can capture the nonlinear mapping between input and output, and automatically learn local area features and high-level abstract features through multi-layer network structures which are usually better than manual extraction and predefined feature sets. Based on deep learning, especially fully convolutional network (FCN) [23] and U-Net [24], have been successfully applied to this field and achieve outstanding performance when compared with conventional approaches. For example, Yap et al. [25] developed several FCN-based variants for the semantic segmentation of breast lesion in BUS images. Hu et al. [26] investigated the effectiveness of Fully Convolutional Networks (FCNs) and U-Net for breast mass segmentation. Almajalid et al. [27] modified and improved U-Net for lesion segmentation based on the contrast-enhanced and speckle-reduced BUS images. However, pattern complexity and intensity similarity between the surrounding tissues (i.e., background) and lesion regions (i.e., foreground) increase the difficulty in lesion segmentation [28], Therefore, the network segmentation performance is required. However, many U-Net and its various variants’ basic architectures now consist of an encoder-decoder structure with a fixed receptive field. Due to the sequential optimization strategy of multi-scale features, the classical U-Net is experiencing the vanishing gradient problem with different optimization issues. Furthermore, the contextual information from multiple scales is not properly converged into the reconstruction of segmentation masks [29]. This might not fully exploit the fusion of coarse-to-fine features from both the encoder and decoder. Moreover, the structures and edges of lesions in BUS images are often blurry, which poses challenges in learning the information related to lesion structures and edges, leading to a decrease in algorithm performance [30].

In order to solve the above problems, we propose a novel variant of the U-Net model, ACL-DUNet, which incorporates dense connections and multiple attention mechanisms (Attention Gates, Channel Attention, and Scale Attention) to improve segmentation accuracy. Our model aims to address the limitations of previous methods by enhancing feature extraction and focusing on relevant regions in the image., the model mainly makes the following three contributions:

• A novel variant of the U-Net model for breast tumor segmentation is proposed:

We have adopted a densely connected CNN structure [31] as the encoder part. This allows each feature extraction layer to receive inputs directly from the feature maps of all previous layers. Not only does this method enable feature reuse, but it also allows for the extraction of breast tumor features of various shapes without adding extra parameters. Moreover, this structure helps to mitigate the problem of vanishing gradients, enhancing the model’s stability and training efficiency.

• Multiple attention mechanisms are integrated in the decoder part:

In the decoder part, we have integrated a CNN with Attention Gates (AGs) [32], Channel Attention [33, 34], and Scale Attention [35]. It has been demonstrated that saliency maps can provide clues/priors by highlighting visually significant regions or objects in images, thereby improving the network’s segmentation performance. For instance, Vakanski et al. [36] proposed integrating the visual saliency of lesion areas into the network, where saliency is introduced through several attention modules. The integration of these attention modules helps achieve more precise segmentation results. During the training process, Attention Gates (AGs) utilize spatial attention to enhance regions of interest on the feature map while suppressing potential background or irrelevant areas. This strengthens the relationships between pixels, enabling the network to better focus on the segmentation target [32]. Channel attention is used within the network to calibrate the cascade of low-level and high-level features, allowing more relevant channels to obtain higher coefficient weights. Feature channels in the encoder primarily contain low-level information, while those in the decoder carry more semantic information. Therefore, they may have different levels of importance for the segmentation task. To better utilize the most informative feature channels, we introduce channel attention to automatically highlight relevant feature channels while suppressing irrelevant ones. Scale Attention Modules are used to capture feature maps at different scales in the backbone network. To better handle objects of varying scales, a reasonable approach is to combine these features for final prediction. However, for a given object, feature maps at different scales might have varying degrees of relevance. The ideal practice is to automatically determine scale-specific weights for each pixel, allowing the network to adapt to the appropriate scale of the given input. Thus, we employ Scale Attention Modules, which automatically learn image-specific weights for each scale to calibrate features at different scales. This module is used at the end of the network [35].

• Performance enhancement and effect validation:

By combining densely connected structures and various attention mechanisms, the newly proposed network architecture outperforms the basic U-Net and other existing methods in the task of breast tumor segmentation. Furthermore, In this study, we evaluated our proposed method on two publicly available breast ultrasound datasets (Mendeley and BUSI) and demonstrated its superior performance compared to existing approaches. validating the effectiveness of the new architecture.

The remainder of this article is organized as follows: In Section 2, the datasets used in this study will be introduced, and a detailed description of the proposed ACL-DUNet will be provided. In Section 3, the experimental setup and results will be presented and analyzed. The discussion and conclusion will be presented in Sections 4 and 5, respectively.

## 2 Method

### 2.1 Network structure

**Fig 1:**
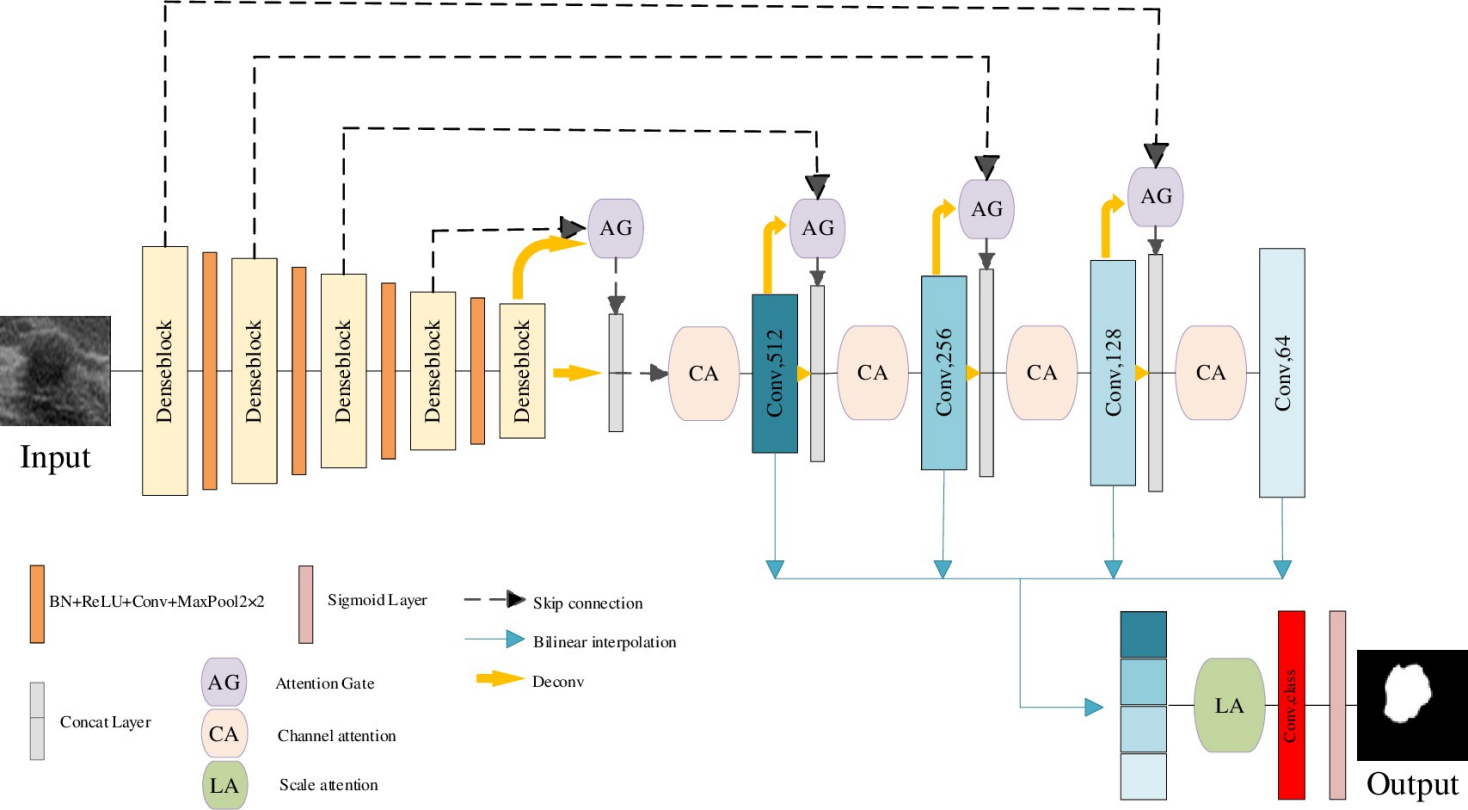
ACL-DUNet network structure diagram.

The datasets used in this work have the following characteristics: a) Different breast tumors exhibit significant variations in size and shape; b) The size of the tumors is relatively small compared to the background. Considering these characteristics, we propose a method based on the encoder-decoder architecture, where the encoder employs a densely connected network, and the decoder incorporates Attention Gates (AGs), Channel Attention (CA), and Scale Attention (LA). The details of using these methods are explained later. The complete structure of the proposed ACL-DUNet is illustrated in Fig 1.

### 2.2 Dense connection networks

Firstly, breast tumors often exhibit variations in shape and size, and the limited availability of training images makes it challenging to segment breast tumors of different shapes and sizes on a small dataset. To address this issue, we adopt the dense connection proposed by G. Huang et al. [31]. This approach connects each layer in a feed-forward manner to all other layers. It means that each layer receives additional inputs from all preceding layers and passes its feature maps to all subsequent layers. This dense connection scheme enables feature reuse, allowing the network to better utilize features and achieve higher segmentation accuracy for small targets with significant scale variations. Additionally, it improves gradient backpropagation, making the network easier to train since each layer can directly receive the error signal from the final output, enabling implicit "deep supervision." Traditional CNN only connects the output feature map of (*l*−1)^*th*^ layer as input to *l*^*th*^ layer. While the *l*^*th*^ layer of a densely connected network receives the characteristic map *x*_0_,*x*_1_,…,*x*_*l*_ of all previous layers, as input:

$$x_{l}=H_{l}(x_{0},x_{1},\ldots ,x_{l})$$ (1)

where [*x*_0_,*x*_1_,…,*x*_*l*_ ] represents the concatenation of the feature maps generates in layers 0,…, *l* − 1. And *H*_*l*_(⋅) is defined as a composite function of three continuous operations: Batch Normalization (BN), ReLU and 3×3 convolution (Conv).

### 2.3 Attention module

**Fig 2:**
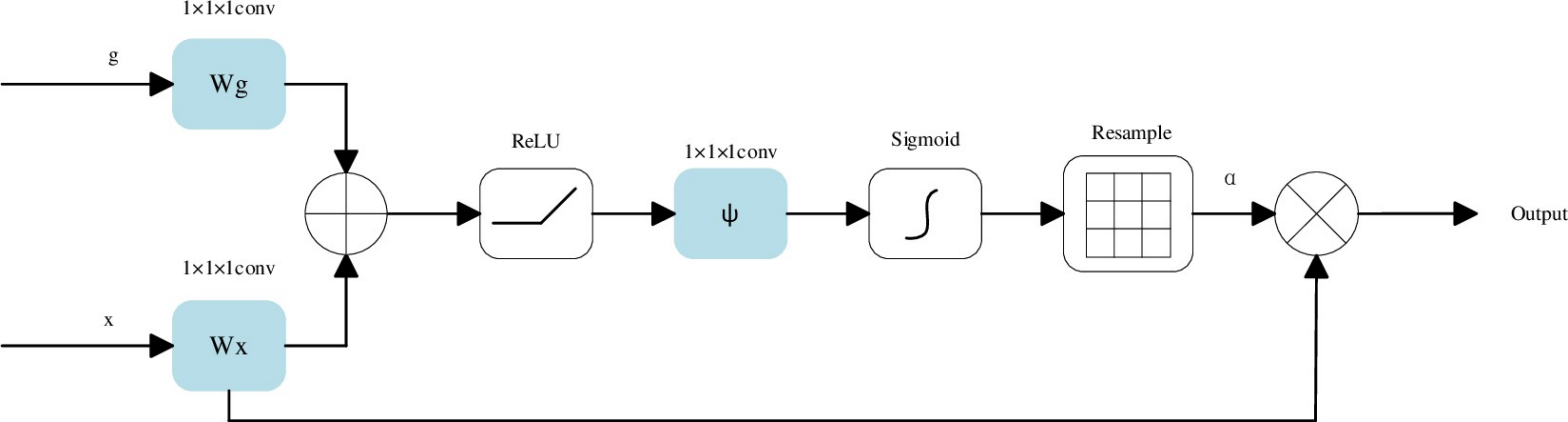
Schematic of additive AGs.

Humans possess a unique visual attention mechanism that allocates more attention to target regions and suppresses irrelevant or distracting information. Therefore, incorporating this attention mechanism in breast tumor segmentation can help the network identify smaller tumor regions in breast ultrasound images compared to the background. The AGs proposed by Oktay [32] fit this attention mechanism, and its main goal is to find the most important information of the current task from a large amount of information, while improving the performance of the network by suppressing task-independent features. The structure of AGs can be seen in Fig 2. *x* is the feature map input from the encoder, and *g* is the feature map from upsampling. *α* is the attention factor generated by the network. The output of AGs is an element-by-element multiplication of the two, as follows:

$$x_{lout}=\alpha \cdot x$$ (2)

If it is in the case of multisemantics, it is necessary to learn the multidimensional attention coefficient.

Our method uses additive attention instead of multiplicative attention to obtain the gate coefficients. Although additive attention introduces some computational overhead, it has been shown to achieve higher accuracy compared to multiplicative attention. The formula for additive attention is as follows:

$$\alpha =\sigma_{2}\left(\psi^{T}(\sigma_{1}\left(W_{x}^{T}x+W_{g}^{T}g+b_{g}\right))+b_{\psi }\right)$$ (3)

*σ*_1_ is often chosen as ReLU function: *σ*_1_(*m*) = max(0,*m*), and *σ*_2_ is the Sigmoid function:$\sigma_{2}(m)=\frac{1}{1+e^{-m}}$, *W*_*x*_, *W*_*g*_ and *ψ* are linear transformations, *b*_*g*_ and *b*_*ψ*_ are bias terms. Parameters of AGs use a normal distribution initialization method and are updated according to the backpropagation principle.

**Fig 3:**
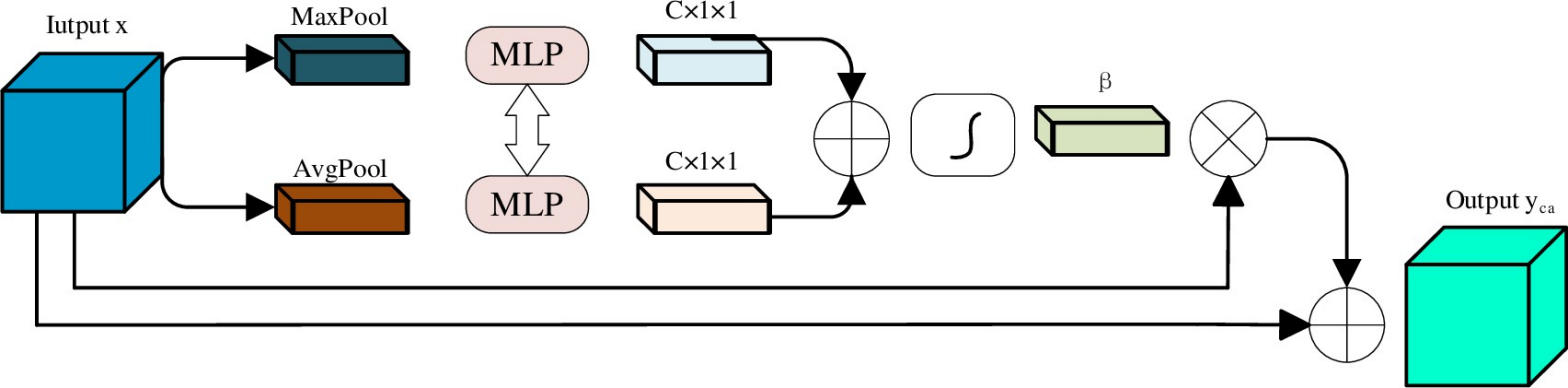
Structure of our proposed channel attention module with residual connection.

In our network, unlike the previous SE block [33] that only used average pooling, we added a max pooling operation to preserve more information [34]. This Channel Attention (CA) module receives both low-level features from the encoder calibrated by AGs and high-level features from the decoder. The feature channels from the encoder contain more fine-grained information, while the feature channels from the decoder contain more coarse-grained information. To better utilize the useful feature channels, we introduced this Channel Attention module. The details of this module are shown in Fig 3.

In our setting, let *x* represent the concatenated input feature map with *C* channels, a global average pooling and a global maximal pooling are first used to obtain the global information of each channel, respectively. A multiple layer perception (MLP) is used to obtain the channel attention coefficient *β*∈[0,1]^*C*×1×1^. The MLP consists of two fully connected layers.

The output of the first layer is *C*/*r*, followed by a ReLU activation function. The second layer has an output channel of *C*. The two results from the MLP are summed and then passed through a Sigmoid activation function to obtain *β*.Our channel attention module output is:

$$y_{ca}=x\cdot \beta +x$$ (4)

**Fig 4:**
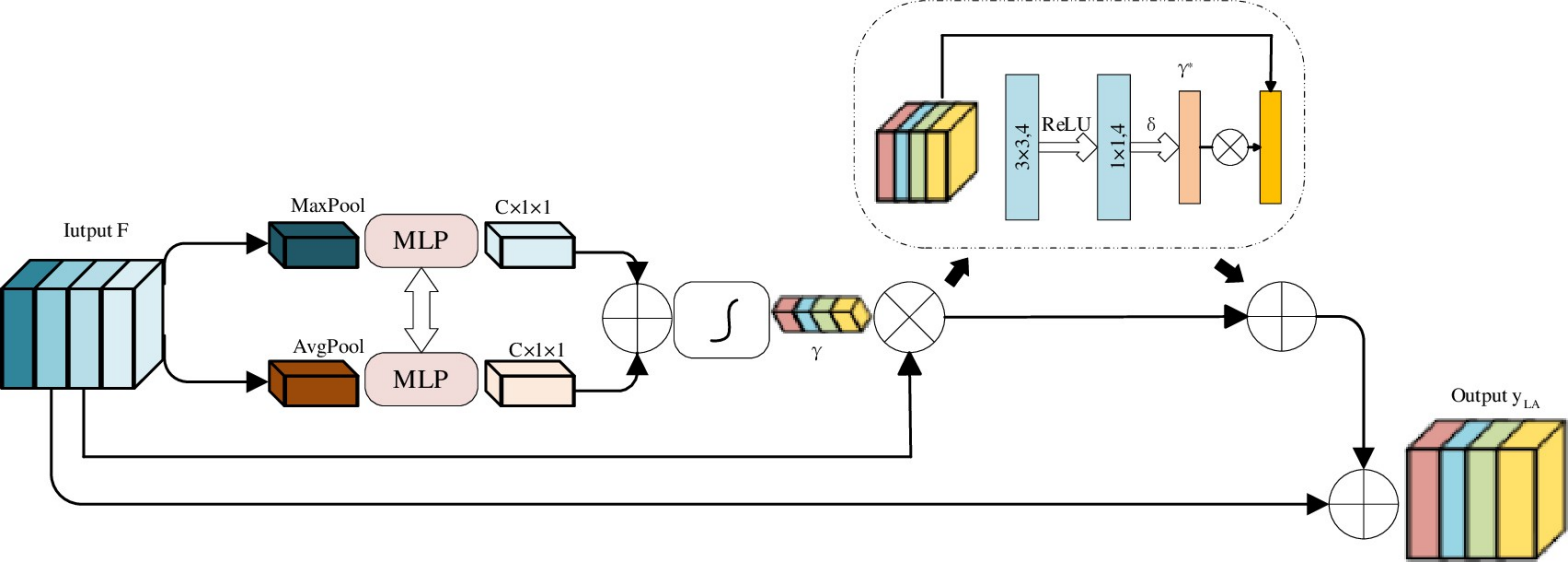
Structure of our proposed scale attention module with residual connection.

The backbone of our network produces feature maps of different scales, and in order to deal with objects at different scales, these feature maps of different scales can be combined and predicted. Ran Gu et al. [35] proposed a new scale attention (LA) module on top of the channel attention module. This module automatically determines the proportional weight of each pixel to calibrate features at different scales so that the network can adapt to the corresponding proportion of a given input image. The structure of the scale attention module is shown in Fig 4.

First of all, the decoder of the network has four layers, so there will be feature maps of four scale sizes, and we use bilinear interpolation to restore these four sizes of feature maps to be as large as the original input image. In order to reduce the amount of computation, a 1×1 convolutional layer is used to compress the feature map into 4 channels, and then the results of compression at different scales are combined into a 16-channel hybrid feature map *F*. Similar to the CA module, the scale attention coefficient is expressed as *γ*∈[0,1]^4×1×1^, In order to assign attention weights on pixels, spatial attention blocks are used *F*⋅*γ* as input to generate spatial directional attention coefficients γ*∈[0,1]^1×*H*×*W*^. So *γ*⋅γ* represents attention on pixels. The final output of the LA module is:

$$y_{LA}=F+F\cdot \gamma +F\cdot \gamma \cdot \gamma^{*}$$ (5)

## 3 Experimental results

In this section, we begin by presenting the experimental setup, including parameter configurations, evaluation strategies and datasets. Subsequently, we provide a detailed account of the experimental results obtained from different methods on two distinct breast ultrasound datasets to validate the effectiveness of the proposed approach.

### 3.1 Dataset description

**Fig 5:**
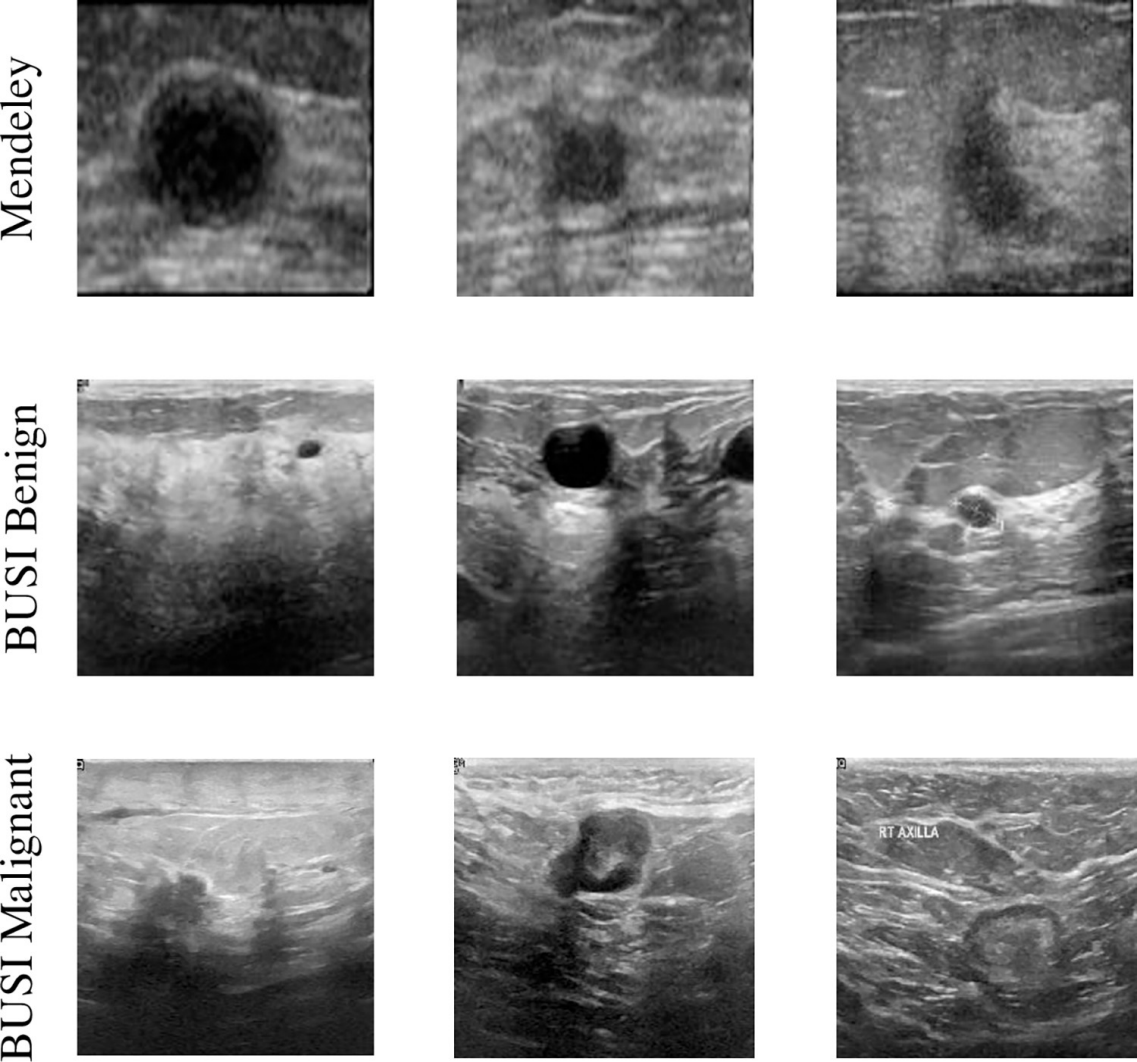
Mendeley dataset images and BUSI dataset benign and malignant pictures.

• Mendeley (Paulo Sergio Rodrigues, 2017) Ultrasound dataset [29, 37] includes 100 benign images and 150 images of malignant cancer. The native resolution of the ultrasound image is 64×64 pixels, which is converted to 128×128 pixels. The dataset is essentially classification-based and does not provide ground truth imagery. Therefore, with the help of experienced radiologists, benign and malignant tumor images are annotated for the model training process. The dataset image is shown in Fig 5.

• The BUSI dataset was provided by Bahya Hospital in Cairo, Egypt (Al Dhabyani et al., 2020) [38]. All ultrasound images were acquired by the LGIQ E9 ultrasound and LGIQ E9 Agile ultrasound systems. The dataset includes images of 133 normal, 487 benign, 210 malignant, for a total of 780 patients. Since the other datasets used in our study included only one breast mass per image, to make later performance comparisons more straightforward, we removed the US images from the BUSI dataset that included multiple breast masses. This modification resulted in 630 US images corresponding to 421 benign and 209 malignant breast masses. Live ground imagery is also provided by radiologists with an image resolution of 1280 × 1024. And the nearest neighbor interpolation method is used to scale the image to 256*256 size. The dataset image is shown in Fig 5.

### 3.2 Experimental setup

The method was implemented using Python 3.6 and the PyTorch deep learning library on an Ubuntu 18.04 system with an NVIDIA TESLA T4 GPU. The images from both datasets were provided in PNG format. For experimentation purposes, the entire dataset was divided into a training dataset (80%) and a testing dataset (20%). And the training process utilized the Adam optimizer with a momentum of 0.9. All parameters were initialized using the "he_normal" method [39]. The initial values for the batch size, learning rate, and number of iterations were set to 8, 0.0001, and 200, respectively. The loss function employed for training was the cross-entropy loss, which calculates the difference between predicted values and ground truth labels. The loss is then propagated through backpropagation to update the weights and biases of the network’s layers.

Before conducting relevant experiments, we also performed a series of data set preprocessing. First, we performed data cleaning, manually checked the two data sets, and eliminated some data that did not meet the requirements. Secondly, the input data was scaled and flipped horizontally and vertically according to probability. Finally, the data was standardized and the mean and standard deviation were set.

### 3.3 Evaluation measures

In this work, we used five metrics to evaluate the performance of the model, namely Dice Similarity Coefficient (DSC), Jaccard Similarity Coefficient (JSC), Positive Predictive Value (PPV), Sensitivity (SEN), and F1-score. The definitions of all evaluation metrics are as follows:

$$DSC=\frac{2\times TP}{2\times TP+FP+FN}$$ (6)

$$JSC=\frac{TP}{TP+FP+FN}$$ (7)

$$PPV=\frac{TP}{TP+FP}$$ (8)

$$SEN=\frac{TP}{TP+FN}$$ (9)

$$F1-score=\frac{2\times PPV\times SEN}{PPV+SEN}$$ (10)

In this context, TP (True Positive) and FP (False Positive) represent the number of correctly predicted tumor pixels inside and outside the ground truth tumor region, respectively. FN (False Negative) and TN (True Negative) represent the number of incorrectly predicted background pixels inside and outside the ground truth tumor region, respectively. Furthermore, all evaluation metrics, including DSC, JSC, PPV, SEN, and F1-score, have a range of 0 to 1, where values closer to 1 indicate better performance. A value of 1 indicates perfect segmentation, meaning the model’s predictions match the ground truth completely, while a value of 0 indicates poor segmentation, indicating no overlap between the model’s predictions and the ground truth.

### 3.4 Experimental results

#### 3.4.1 Ablation studies

**Fig 6:**
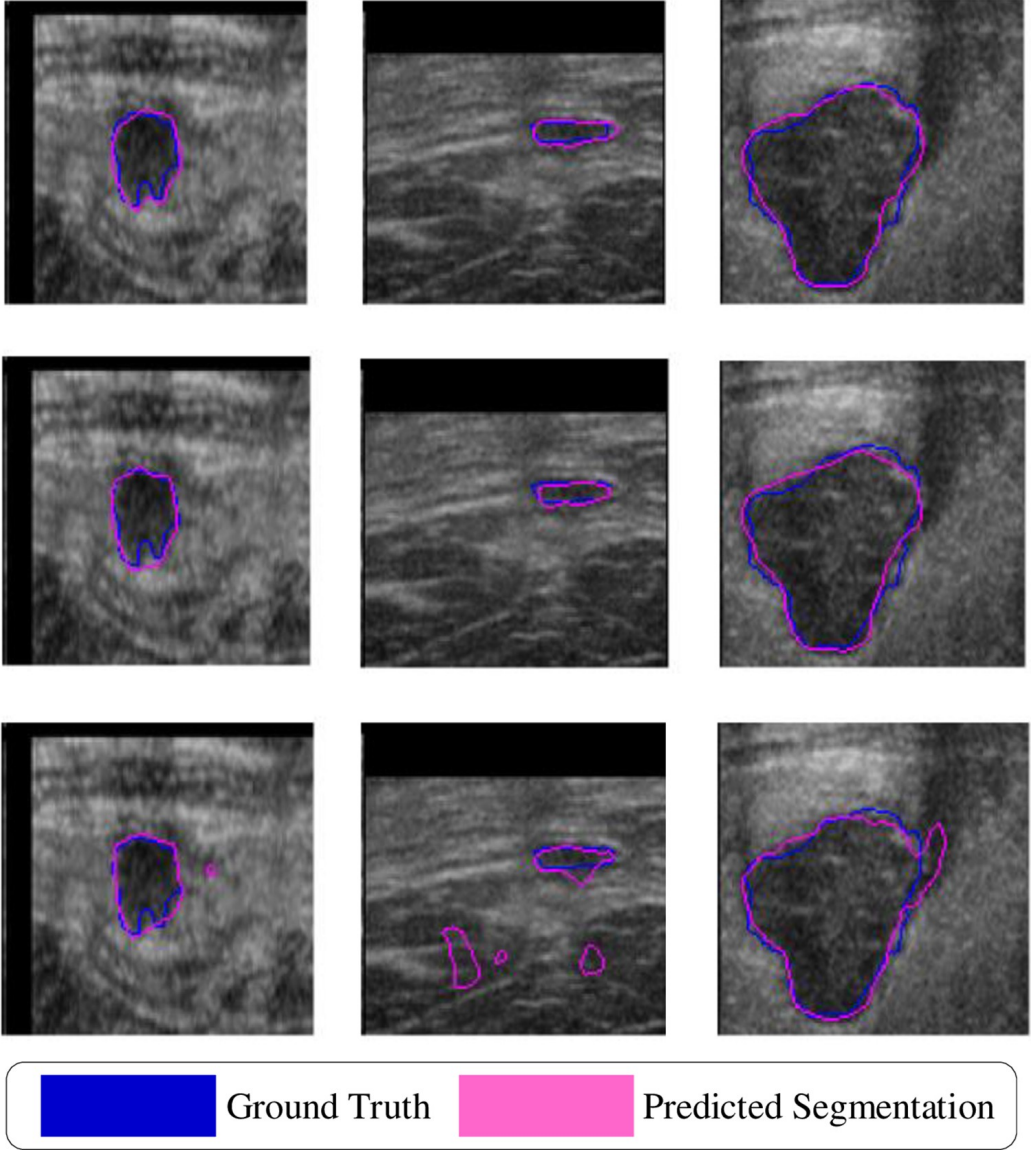
In the ablation experiment, Mendeley data segmentation plot. Blue is Ground Truth and pink is Prediction segmentation.

Table 1 shows the results of the networks with and without Dense Connections (DC) and the three attention modules (ACL) on the Mendeley dataset. On the original dataset, the network with only DC achieved average results of DSC (0.8270), JSC (0.7169), PPV (0.9255), SEN (0.7613), and F1 (0.8354). The network with only the ACL modules achieved average results of DSC (0.8646), JSC (0.7669), PPV (0.9339), SEN (0.8109), and F1 (0.8680). In contrast, the ACL-DUNet, which combines DC and ACL, achieved the following average scores on each evaluation metric: DSC (0.8764), JSC (0.7807), PPV (0.9482), SEN (0.8152), and F1 (0.8767). Compared to the network with only DC, ACL-DUNet showed significant improvements in DSC (4.94%), JSC (6.38%), PPV (2.27%), SEN (5.39%), and F1 (4.13%). Moreover, when compared to the network with only ACL, ACL-DUNet also demonstrated improvements in DSC (1.18%), JSC (1.38%), PPV (1.43%), SEN (0.43%), and F1 (0.87%) on all five evaluation metrics. The segmentation results on this dataset are shown in Fig 6.

**Table 1:** Ablation experiments on the Mendeley dataset.

Method	DC	ACL	DSC↑	JSC↑	PPV↑	SEN↑	F1↑
ACL-DUNet	√	×	0.8270	0.7169	0.9255	0.7613	0.8354
ACL-DUNet	×	√	0.8646	0.7669	0.9339	0.8109	0.8680
ACL-DUNet	√	√	**0.8764**	**0.7807**	**0.9482**	**0.8152**	**0.8767**

**Fig 7:**
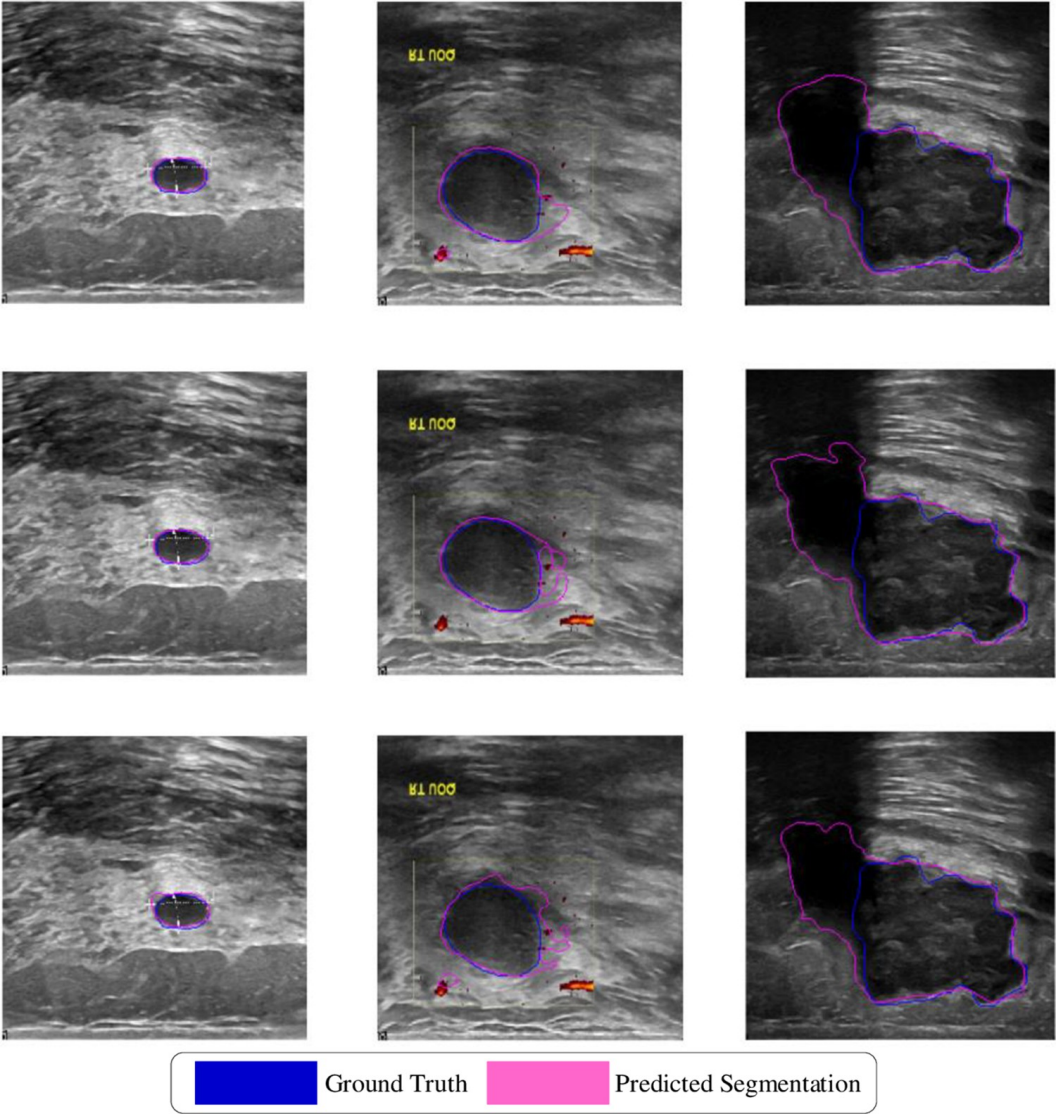
In the ablation experiment, BUSI data segmentation plot. Blue is Ground Truth and pink is Prediction segmentation.

Table 2 presents the results of the networks with and without Dense Connections (DC) and the three attention modules (ACL) on the BUSI dataset. On the BUSI dataset, the network with only DC achieved average results of DSC (0.7495), JSC (0.6569), PPV (0.8001), SEN (0.7564), and F1 (0.7776). The network with only the ACL modules achieved average results of DSC (0.8111), JSC (0.7152), PPV (0.8157), SEN (0.8660), and F1 (0.8401). In contrast, the ACL-DUNet, which combines DC and ACL, achieved the following average scores on each evaluation metric: DSC (0.8313), JSC (0.7344), PPV (0.8292), SEN (0.8703), and F1 (0.8492). Compared to the network with only DC, ACL-DUNet showed improvements in DSC (8.18%), JSC (7.75%), PPV (2.91%), SEN (11.39%), and F1 (7.16%). Moreover, when compared to the network with only ACL, ACL-DUNet also demonstrated improvements in DSC (2.02%), JSC (1.92%), PPV (1.35%), SEN (0.43%), and F1 (0.91%) on all five evaluation metrics. The segmentation results on this dataset are shown in Fig 7.

**Table 2:** Ablation experiments on the BUSI dataset.

Method	DC	ACL	DSC↑	JSC↑	PPV↑	SEN↑	F1↑
ACL-DUNet	√	×	0.7495	0.6569	0.8001	0.7564	0.7776
ACL-DUNet	×	√	0.8111	0.7152	0.8157	0.8660	0.8401
ACL-DUNet	√	√	**0.8313**	**0.7344**	**0.8292**	**0.8703**	**0.8492**

Some challenging visual results are depicted in Figs 6 and 7. Fig 6 shows the results of the ablation experiment on the Mendeley dataset. The first row contains three segmentation images from ACL-DUNet with both Dense Connections (DC) and Attention modules (ACL). The second row shows the segmentation images from ACL-DUNet with only ACL modules, and the third row displays the segmentation images from ACL-DUNet with only DC. The blue lines represent the Ground truth, and the pink lines represent the Predicted Segmentation. It can be observed that in the first row, the second and third images exhibit the best segmentation performance among the ablation experiments, while the first image performs similarly to the second row’s images. On the other hand, the segmentation results of ACL-DUNet with only DC (third row) consistently perform the worst, which aligns with the findings in Table 1. Fig 7 presents the results of the ablation experiment on the BUSI dataset. Similarly, the first row contains three segmentation images from ACL-DUNet with both DC and ACL, the second row shows the segmentation images from ACL-DUNet with only ACL modules, and the third row displays the segmentation images from ACL-DUNet with only DC. The blue lines represent the Ground Truth, and the pink lines represent the Predicted Segmentation. It can be observed that the first and second images in the first row show excellent segmentation performance, closely approximating the Ground Truth. The second image slightly exhibits over-segmentation, but the overall performance is still relatively close to the Ground Truth, especially in the surrounding areas of the tumor segmentation. However, the segmentation results for malignant tumors (third column) are not satisfactory in all cases. These results provide visual evidence that the proposed ACL-DUNet with both Dense Connections and Attention modules achieves superior segmentation performance on both datasets compared to the ablation experiments with only one of the components.

#### 3.4.2 Comparison with other methods

**Fig 8:**
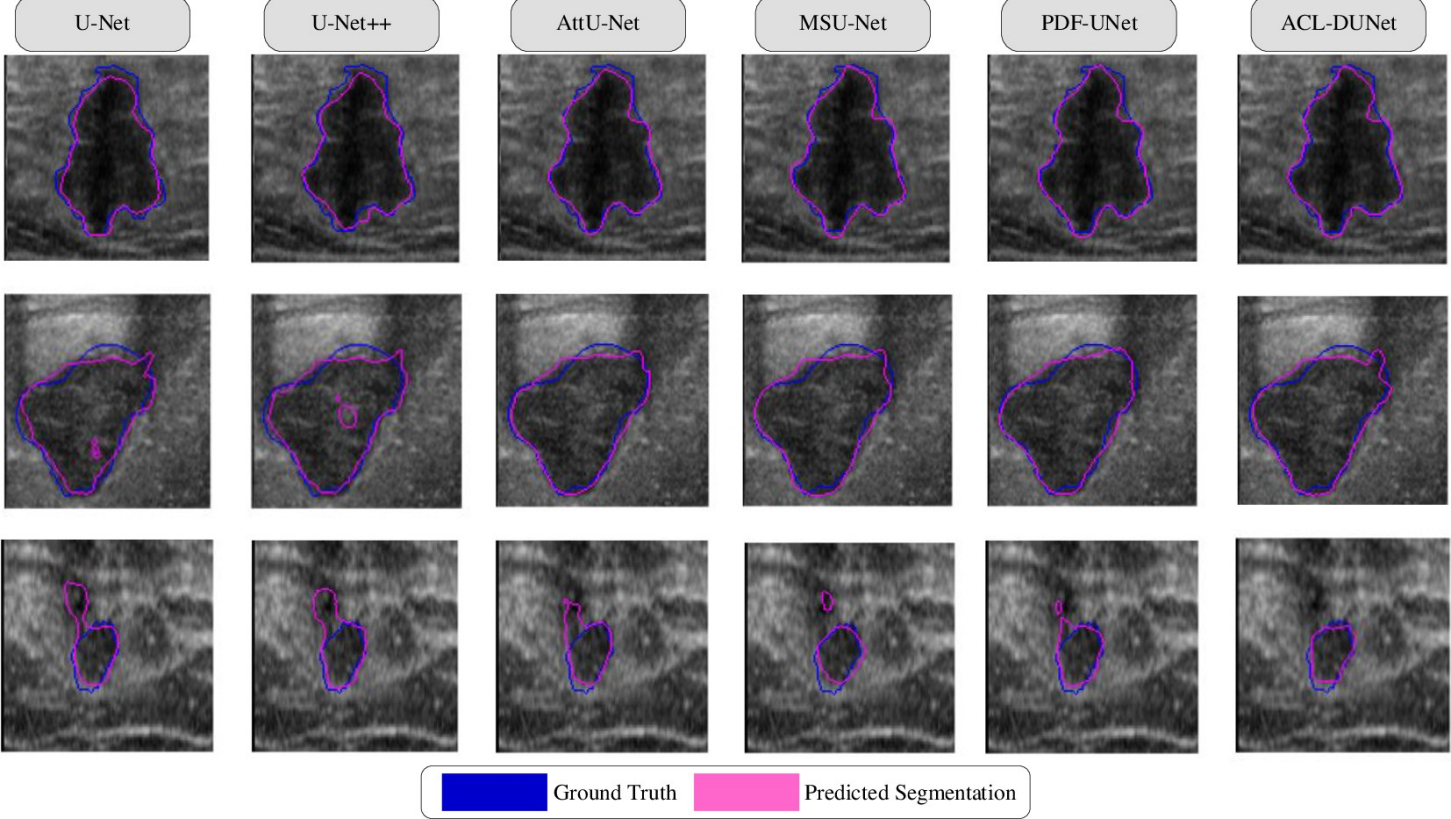
On the Mendeley dataset, our method is a segmentation plot of the method versus the contrast method.

Table 3 presents the performance of ACL-DUNet on the Mendeley dataset compared to other state-of-the-art methods [24, 29, 32, 40, 41]. It can be observed that our network outperforms all the compared methods in all five evaluation metrics. On the Mendeley dataset, ACL-DUNet achieves a 5.35% improvement in DSC measurement, a 6.83% improvement in JSC measurement, a 4.88% improvement in PPV measurement, a 4.29% improvement in SEN measurement, and a 4.57% improvement in F1 measurement compared to U-Net. The second-best results are observed in PDF-Unet, with a DSC measurement of 0.8574, a JSC measurement of 0.7594, a SEN measurement of 0.7946, and an F1 measurement of 0.8638. Additionally, the second-best PPV measurement is achieved by the AttU-Net, with a PPV measurement of 0.9466. On the other hand, the worst-performing network in all five metrics is U-Net++, with measurements of DSC (0.8118), JSC (0.7001), PPV (0.8747), SEN (0.7700), and F1 (0.8190). The segmentation results of these methods on images can be seen in Fig 8.

**Table 3:** Control experiments on the Mendeley dataset.

Method	DSC↑	JSC↑	PPV↑	SEN↑	F1↑
U-Net [24]	0.8229	0.7124	0.8994	0.7723	0.8310
U-Net++ [40]	0.8118	0.7001	0.8747	0.7700	0.8190
AttU-Net [32]	0.8526	0.7492	0.9466	0.7760	0.8529
MSU-Net [41]	0.8475	0.7453	0.9340	0.7841	0.8525
PDF-Net [29]	0.8574	0.7594	0.9462	0.7946	0.8638
ACL-DUNet	**0.8764**	**0.7807**	**0.9482**	**0.8152**	**0.8767**

**Fig 9:**
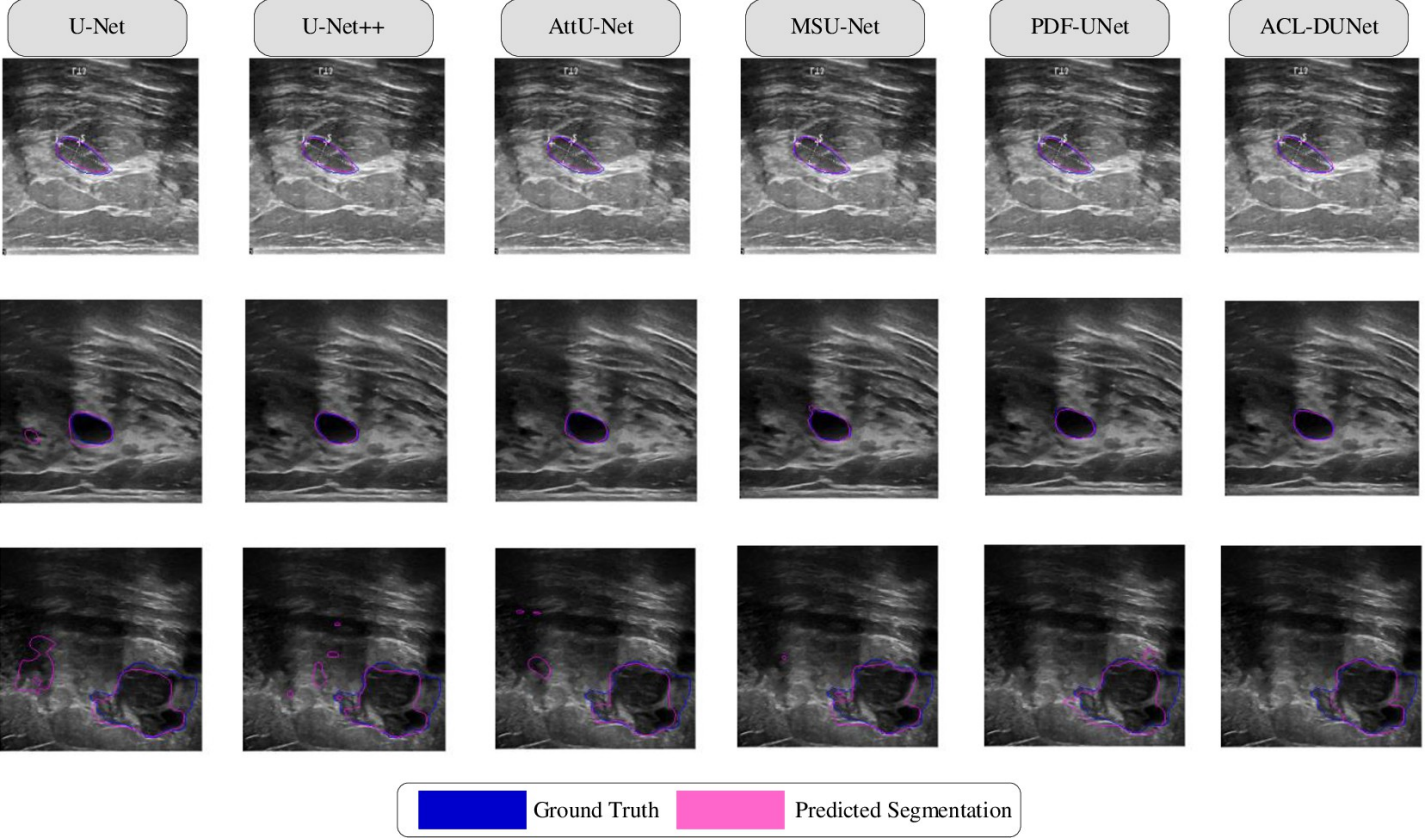
On the BUSI dataset, our method is a segmentation plot of the method versus the contrast method.

Table 4 illustrates the performance of ACL-DUNet on the BUSI dataset compared to other state-of-the-art methods. It can be observed that our network outperforms all the compared methods in overall performance. On the BUSI dataset, ACL-DUNet achieves a 9.31% improvement in DSC measurement, a 10.06% improvement in JSC measurement, a 13.38% improvement in SEN measurement, and a 6.81% improvement in F1 measurement compared to U-Net. Although there is a slight 0.22% decrease in PPV, considering the improvement in F1, it falls within an acceptable range. The second-best overall results are observed in MSU-Net, with a DSC measurement of 0.8055, a JSC measurement of 0.7175, a PPV measurement of 0.8573, a SEN measurement of 0.8140, and an F1 measurement of 0.8351. Additionally, MSU-Net achieves the best PPV measurement among all six networks, with a PPV measurement of 0.8573. On the other hand, U-Net performs the worst in overall performance among the five metrics, with measurements of DSC (0.7382), JSC (0.6338), PPV (0.8314), SEN (0.7365), and F1 (0.7811). The segmentation results of these methods on images can be seen in Fig 9.

**Table 4:** Control experiments on the BUSI dataset.

Method	DSC↑	JSC↑	PPV↑	SEN↑	F1↑
U-Net [24]	0.7382	0.6338	0.8314	0.7365	0.7811
U-Net++ [40]	0.7526	0.6489	0.8173	0.7546	0.7847
AttU-Net [32]	0.7487	0.6416	0.8137	0.7651	0.7886
MSU-Net [41]	0.8055	0.7175	**0.8573**	0.8140	0.8351
PDF-Net [29]	0.7810	0.6862	0.8471	0.7844	0.8145
ACL-DUNet	**0.8313**	**0.7344**	0.8292	**0.8703**	**0.8492**

Some challenging visual results are depicted in Figs 8 and 9. Fig 8 shows the segmentation results of the ablation experiment on the Mendeley dataset. The blue lines represent the Ground Truth, and the pink lines represent the Predicted Segmentation. It can be observed that the segmentation results of all models in the first row are quite good. In the second row, there is a noticeable difference in segmentation between the first two images, while the following four images have similar performance, with PDF-Unet showing smoother predicted lines. In the third row, the segmentation results of ACL-DUNet are not as prominent, indicating its superior performance. Fig 9 displays the segmentation results of the ablation experiment on the BUSI dataset. Similarly, in the first row, there is not much difference in performance among all models, but MSU-Net and ACL-DUNet have closer alignment between the blue and pink lines. In the second row, ACL-DUNet shows almost complete overlap with the Ground Truth, indicating its superior performance, followed by PDF-Unet. In the third row, the segmentation of malignant tumors is challenging, and none of the models achieve complete segmentation.

## 4 Discussion

Breast cancer is a major cause of cancer-related death among women worldwide, and the automatic segmentation of breast masses in ultrasound images is of significant clinical importance as it helps doctors detect early signs of breast cancer. In the task of breast tumor segmentation, the targets may exhibit substantial variations in position, texture, shape, and scale, making the awareness of spatial sizes and positions crucial for any network to achieve accurate segmentation. In this study, we explored a novel approach for breast ultrasound image segmentation, referred to as ACL-DUNet. This method combines the U-Net architecture with dense connections, attention gates (AGs), channel attention, and scale attention. The dense connected CNN structure is utilized in the down-sampling stage of the network as a feature extractor, allowing for feature reuse and extraction of various shapes of breast tumor features without increasing the number of parameters. It also helps alleviate the vanishing gradient problem. The attention gates (AGs), channel attention, and scale attention are placed in the decoder part of the network. These attention modules aid in obtaining more accurate segmentation results. AGs enhance the regions of interest on the feature maps while suppressing potential background or irrelevant regions. Channel attention is used to calibrate the concatenation of low-level and high-level features in the network, giving higher weights to more relevant channels and highlighting the most useful feature channels while suppressing irrelevant ones. The scale attention aims to better integrate the original semantic predictions obtained from the decoder, and therefore, it is placed at the end of the network.

In the evaluation of the two public datasets, our proposed ACL-DUNet achieved excellent segmentation scores. In the ablation study, the network combining Dense Connection (DC) and ACL outperformed networks with only DC or ACL, ranking first in all five evaluation metrics. Tables 3 and 4 present a comparison between our ACL-DUNet and other research methods on the Mendeley and BUSI datasets. On the Mendeley dataset, our model achieved the highest scores in all five evaluation metrics. Similarly, on the BUSI dataset, our method outperformed others in DSC, JSC, SEN, and F1 metrics. The overall results indicate that our proposed approach can effectively handle breast ultrasound tumor segmentation tasks, enabling clear diagnosis of breast cancer with the highest accuracy.

Despite achieving good performance in this study, there are still some areas for improvement and optimization in the ACL-DUNet. For instance, optimizing the dense connection network to enhance the training speed would be beneficial. Additionally, leveraging post-processing methods, such as Conditional Random Fields, could further improve the automatic segmentation results.

This study also has certain limitations. Firstly, there are limitations related to the datasets used; the model was trained and tested on two relatively small breast ultrasound datasets. Small-scale datasets may limit the model’s ability to generalize to larger or more diverse data sets. Although the model performs well on these small datasets, it may experience a decline in performance in actual clinical applications. Secondly, there is insufficient data diversity; the datasets used may not cover all types of breast tumors, especially rare or atypical tumors. This could lead to insufficient model recognition capabilities for unseen tumor types. At last, the model was not validated in real-time image with uncontrolled settings, model not tested in real-world situation or compared with fellowship trained or experienced annotator restricting its widespread applicability, and source of training biases.

Next, there are limitations regarding the model structure. Firstly, the introduction of multiple attention mechanisms (spatial, channel, and scale attentions) has improved segmentation accuracy but has also increased the model’s complexity and computational cost. Deploying such models may be impractical in resource-constrained environments (such as certain clinical devices). Secondly, there are flexibility constraints; the optimization and performance of the model heavily rely on a specific network architecture (such as variants of U-Net). This design may have limited adaptability when faced with new problems that require different architectural features.

Additionally, there are limitations related to hyperparameters and training details. Firstly, model performance depends on the setting of hyperparameters, such as learning rate, batch size, and regularization coefficients. Optimal values for these parameters are typically obtained through repeated experimentation and may no longer be optimal on different datasets or with updated data. Secondly, despite the use of batch normalization and attention mechanisms, the training process of the model might still face issues with instability and difficulty in convergence, especially in cases with large parameter spaces and multiple layers.

Lastly, there are practical challenges in the technological application. Although the model performs well on public datasets, it lacks extensive validation in actual clinical settings. Differences in breast ultrasound images due to varying equipment and operating conditions may lead to decreased model performance.

## 5 Conclusion

In this study, we propose a densely connected U-Net model that integrates three different attention mechanisms: spatial attention gates, channel attention, and scale attention, for precise segmentation of tumors in breast ultrasound images. Through extensive testing on two publicly available breast ultrasound datasets, our approach has demonstrated the following major achievements and breakthroughs:

1. Improvement in Segmentation Accuracy: Our model has achieved superior segmentation accuracy on the Mendeley and BUSI datasets compared to existing state-of-the-art methods. Specifically, compared to traditional U-Net and other benchmark models, ACL-DUNet has shown improvements in both the Dice Similarity Coefficient (DSC) and Jaccard Similarity Index (JSC), demonstrating its outstanding performance.

2. Effective Integration of Multiple Attention Mechanisms: By integrating spatial attention gates, channel attention, and scale attention, the model more finely captures and utilizes spatial, channel, and scale information of images, enhancing the distinction between tumor regions and the background. This integrated approach not only boosts the model’s feature extraction capabilities but also optimizes the flow of information across the network.

3. Robustness and Generalization Capability: ACL-DUNet has proven its efficiency and accuracy in handling tumors of various sizes and shapes, maintaining high segmentation performance even in cases of variable image quality and indistinct tumor presentations. Additionally, the model has demonstrated good generalization across two different datasets, indicating its adaptability to various clinical settings and scanning equipment.

4. Computational Efficiency: Despite the model’s complex structure, through algorithm optimization and network design, ACL-DUNet achieves high accuracy while maintaining relatively fast computation speeds. This is particularly important for clinical applications as it supports rapid diagnostics, helping to improve medical efficiency.

5. Facilitating Clinical Diagnosis and Early Intervention: By providing high-precision tumor segmentation, this model can assist radiologists in accurately identifying and classifying breast tumors at early stages, thereby improving the diagnostic and treatment processes for patients. This is crucial for enhancing the survival rates and quality of life of breast cancer patients.

In summary, our study proposes a densely connected U-Net model with integrated attention mechanisms for precise segmentation of breast tumors in ultrasound images. Through extensive testing on two publicly available datasets, our model has demonstrated significant improvements in segmentation accuracy, feature extraction, and computational efficiency.

However, future research should focus on validating the model on larger and more diverse datasets, simplifying the model structure, optimizing training stability, and conducting extensive clinical validations. By addressing these limitations, our model can further enhance its applicability and effectiveness in clinical settings, ultimately improving breast cancer diagnosis and treatment.
